# Mitochondrial presequences are more than just address labels

**DOI:** 10.1002/pro.70491

**Published:** 2026-02-12

**Authors:** Erik Marcel Heller, Svenja Lenhard, Doron Rapaport, Johannes Herrmann

**Affiliations:** ^1^ Cell Biology, University of Kaiserslautern, RPTU Kaiserslautern Germany; ^2^ Interfaculty Institute of Biochemistry, University of Tübingen Tübingen Germany

**Keywords:** chaperones, mitochondria, Presequence, proteasome, protein import, ubiquitin ligases

## Abstract

Most mitochondrial proteins are synthesized in the cytosol as precursor proteins with N‐terminal presequences. These presequences serve as targeting signals that facilitate the binding to mitochondrial surface receptors and translocation across the mitochondrial membranes. However, recent studies showed that presequences can be more than address tags. They can contain degradation signals recognized by components of the ubiquitin‐proteasome system, and therefore, serve as timers that determine the lifespan of newly synthesized precursor proteins. Moreover, presequences can interact with components of the cytosolic chaperone system to prevent or delay precursor folding. Finally, presequences of some dually localized proteins contain targeting information not only for mitochondria but also for other cellular destinations such as the nuclear lumen or chloroplasts in plant cells. Thus, presequences contain multifaceted information to endow mitochondrial precursor proteins with specific properties that are critical for the early steps of mitochondrial protein biogenesis.

## 1 INTRACELLULAR PROTEIN SORTING, THE CONCEPT OF TARGETING SEQUENCES

Eukaryotic cells are compartmentalized. Since all proteins (except for some organellar translation products) are synthesized in the cytosol, many newly synthesized proteins have to be targeted to their specific cellular location. Targeting signals serve as address tags that are recognized by receptors on the surface of their respective compartment. Examples of such targeting signals include signal sequences on proteins of the endoplasmic reticulum (ER) and the secretory pathway, mitochondrial targeting signals (MTS) and other internal signals on mitochondrial proteins, transit peptides on chloroplast proteins, peroxisomal targeting signals (PTS) on peroxisomal proteins, and nuclear localization signals (NLS) on nuclear proteins (von Heijne, 1994; Wickner & Schekman, 2005). Except for type 1 PTS, these targeting signals have no defined sequence consensus but rather share specific structural or physicochemical properties that characterize each type of signal. Prediction programs make use of these characteristics to identify and classify a large portion of the different signals, often with high precision (Almagro Armenteros et al., 2019). Proteins, which are threaded into their target compartment in an unfolded linear fashion, normally use N‐terminal targeting signals so that their translocation can start before translation is completed. Such N‐terminal targeting signals are characteristic of proteins of the ER, mitochondria, and chloroplasts.

## 2 STRUCTURAL PROPERTIES OF MITOCHONDRIAL PRESEQUENCES

Most mitochondrial proteins are made with an N‐terminal presequence. Presequences of mitochondrial proteins from fungi and animals share the same structural features and are generally exchangeable, suggesting that the general properties of MTSs are conserved throughout these kingdoms. In plant cells, the presence of chloroplasts has influenced the protein targeting situation, as the targeting sequences have to distinguish both bacteria‐derived organelles. In plants, some mitochondrial presequences direct proteins specifically to mitochondria, whereas dually localized proteins have to be recognized both by receptors on mitochondria and on chloroplasts (Pines et al., 2024).

**FIGURE 1:**
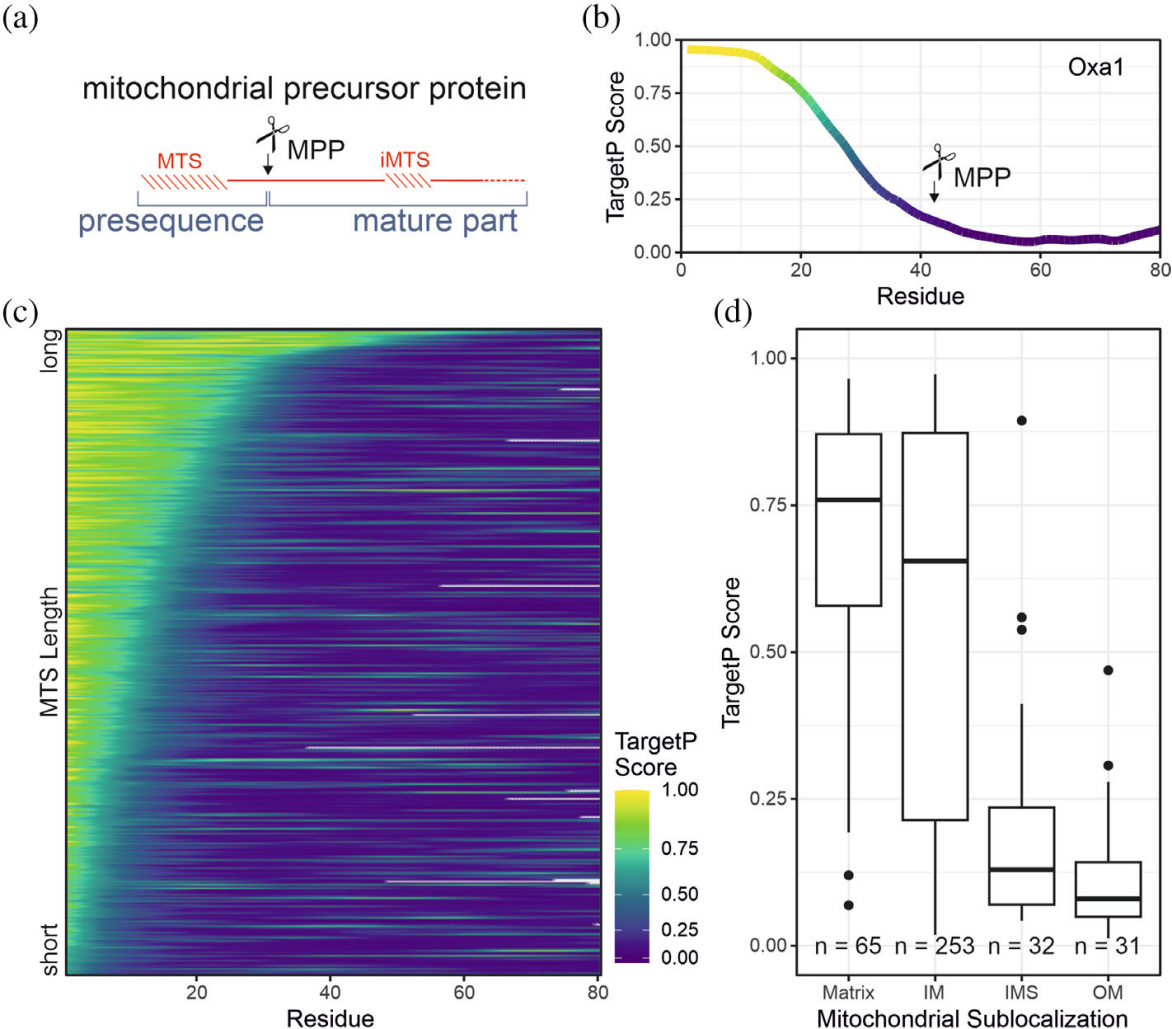
Mitochondrial presequences share physico‐chemical properties. (a) Schematic representation of a mitochondrial precursor protein. MPP, mitochondrial processing peptidase; MTS, mitochondrial targeting signal; iMTS, internal MTS. (b) The TargetP 1.1 scores (Emanuelsson et al., 2000) were calculated for the 80 N‐terminal residues of yeast Oxa1, smoothed by Savitsky‐Golay filtering, and plotted (Boos et al., 2018; Savitzky & Golay, 1964). The position of the MPP cleavage site is indicated. (c) The TargetP 1.1 scores for the 80 N‐terminal residues of all mitochondrial proteins of baker's yeast with a predicted MTS were calculated, smoothed by Savitsky‐Golay filtering, and sorted by length of the predicted MTS. Proteins of yeast mitochondria (Morgenstern et al., 2017) were analyzed with TargetP (Emanuelsson et al., 2007); only proteins with a TargetP score of the N‐terminal residue larger than 0.7 were considered. Proteins were sorted by the length of the MTS, assuming that the MTS ended where the TargetP score fell below 0.7. (d) TargetP scores of proteins of different mitochondrial sub‐compartments. IM, inner membrane; IMS, intermembrane space; OM, outer membrane.

In most, but not in all cases, presequences are proteolytically removed upon translocation into the matrix by the mitochondrial processing peptidase (MPP; Figure 1a). Presequences are typically between 10 and 70 amino acid residues in length and of amphipathic nature with one hydrophobic and one positively charged face (Roise et al., 1986; von Heijne, 1986). They are rich in serine and threonine and lack negatively charged residues. MPP recognizes these properties and cleaves downstream of an arginine residue (R‐2 or R‐3; Habib et al., 2007; Schneider et al., 1998; Vögtle et al., 2009). These common properties allow a reliable prediction of mitochondrial targeting properties in proteins by platforms such as TargetP (Emanuelsson et al., 2000; Emanuelsson et al., 2007), MitoFates (Fukasawa et al., 2015), MitoProt (Claros, 1995) or Predotar (Small et al., 2004). The different prediction programs were described and compared in depth in previous articles (Almagro Armenteros et al., 2019; Emanuelsson et al., 2007; Habib et al., 2007; Martelli et al., 2021; Sun & Habermann, 2017).

In our experience, the TargetP 1.1 tool (Emanuelsson et al., 2000) is particularly useful for the analysis of proteins of baker's yeast (*Saccharomyces cerevisiae*), as this machine learning software was trained on yeast proteins. This algorithm can even be used to predict import‐promoting segments within the mature part of mitochondrial proteins, which were termed internal mitochondrial targeting signals (iMTS; Boos et al., 2018; Jung et al., 2024; Schneider et al., 2021). Such internal signals facilitate the binding of precursor proteins to surface receptors on the mitochondria, preferentially to Tom70 (Backes et al., 2018). Calculation of the TargetP score for each residue in a protein's sequence can be used to identify potential MTS‐like regions in mitochondrial precursor proteins. Figure 1b shows, as an example, a TargetP profile of the N‐terminal 80 amino acid residues of the yeast mitochondrial protein Oxa1. The strong heterogeneity in the lengths of the MTSs becomes obvious when the TargetP values of the N‐terminal 80 amino acid residues of all proteins of yeast mitochondria are plotted (Figure 1c). Most proteins of the matrix and inner membrane have high TargetP values (measured for the start methionine), whereas most IMS and outer membrane proteins lack presequences and MTSs (Figure 1d).

The terms presequence and MTS are often used synonymously, but in sensu stricto, presequences are the N‐terminal regions that are removed by MPP, which typically contain the targeting information for mitochondrial proteins; thus, the MTS.

## 3 FUNCTIONS OF MITOCHONDRIAL PRESEQUENCES

MTSs are both necessary and sufficient for mitochondrial targeting, so that the deletion of the presequence normally impedes the translocation into the mitochondria, and fusion of a presequence to the N‐terminus of a passenger protein usually leads to its import into mitochondria. However, the targeting efficiency can considerably differ: some presequences require highly energized mitochondria, whereas others are less finicky (Vowinckel et al., 2015). Apparently, presequences not only strongly differ in length but also in “strength”, and longer presequences were often found to be more efficient (Rödl et al., 2025; Vögtle et al., 2009; Yan et al., 2025). The molecular features underlying these differences in targeting efficiency are not well understood and might be complex, as they presumably affect the biogenesis of the proteins on different levels. The different functions of presequences will be discussed in the following sections.

## 4 PRESEQUENCES ARE ADDRESS LABELS AND BIND PRESEQUENCE RECEPTORS

**FIGURE 2:**
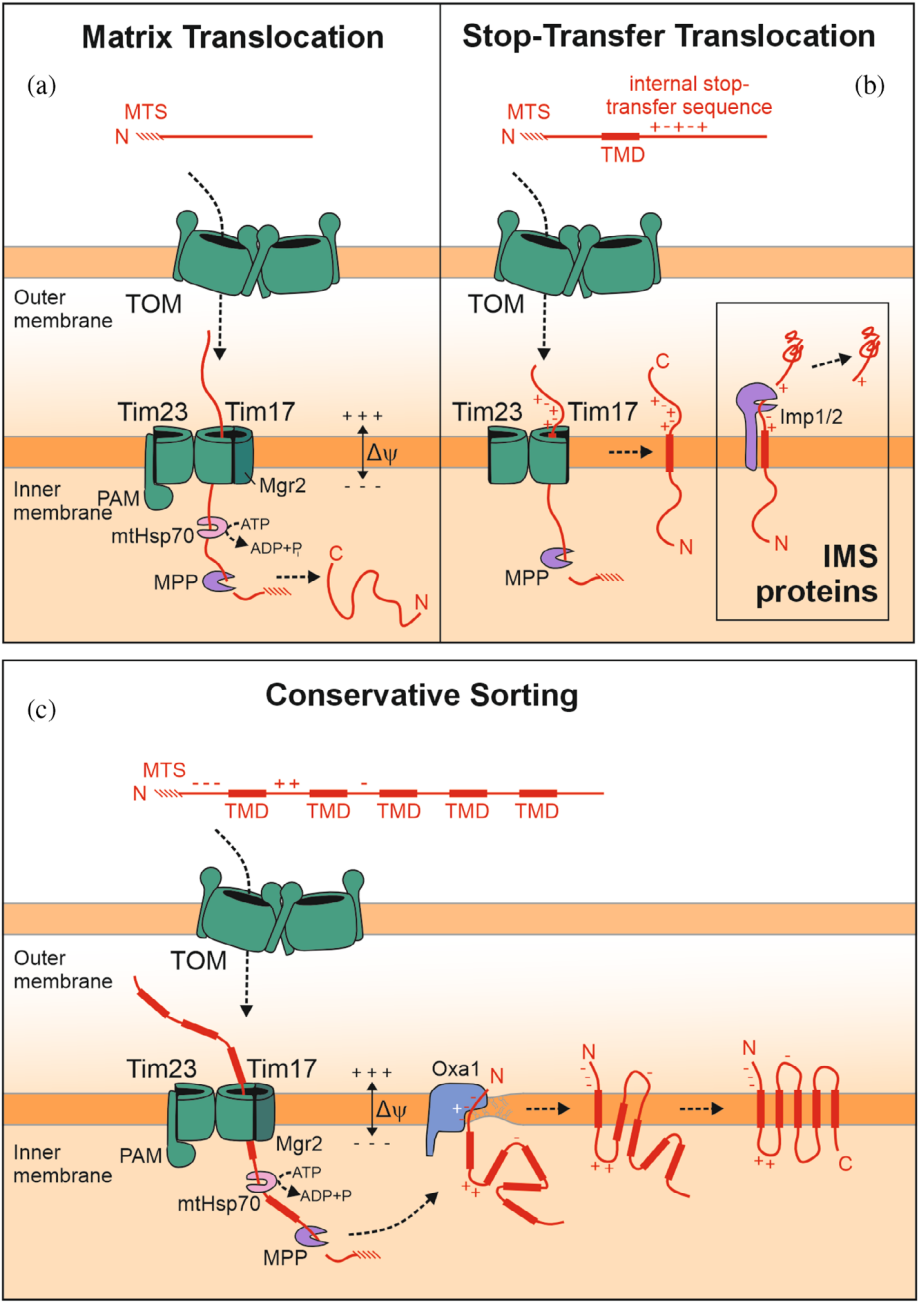
Import routes of presequence‐containing proteins. (a) Schematic representation of the import pathway into the mitochondrial matrix. (b) Proteins of the inner membrane and the IMS contain stop‐transfer signals that facilitate their lateral insertion from the TIM23 complex into the inner membrane. This release might be followed by processing by the inner membrane protease (Imp) 1 which then releases a soluble protein into the IMS. (c) Conservatively sorted inner membrane proteins are inserted from the matrix by the Oxa1 insertase.

Presequences direct proteins from the cytosol into the mitochondrial matrix (Figure 2). Three groups of mitochondrial proteins rely on presequences for their proper biogenesis: (i) those that are destined to the matrix, (ii) proteins that contain a stop‐transfer segment downstream of the presequence and are released laterally into the inner membrane, and (iii) precursor proteins that cross both membranes via the TOM and TIM23 complexes but then are sorted from the matrix to the inner membrane in the conservative sorting pathway that is facilitated by Oxa1 as an insertase (Figure 2).

The presequences of all these proteins interact sequentially throughout their import pathways with a series of mitochondrial binding sites (Dietmeier et al., 1997; Komiya et al., 1998; Mayer et al., 1995). First, after or already during the synthesis of the respective protein, presequences can interact with cytosolic factors that will keep the protein in an import‐competent conformation. Next, the presequence will be recognized by the cytosol‐exposed substrate binding sites of the mitochondrial surface receptors such as Tom20, Tom22, and Tom70 (the so‐called “cis site”; Abe et al., 2000; Komiya et al., 1997; Ramage et al., 1993; Söllner et al., 1990). Then, the presequence will be relayed to the protein‐conducting channel of Tom40 (Araiso et al., 2019; Zhou et al., 2023). Upon emerging from the TOM pore, presequences interact with the IMS domain of Tom22, Tom40, and Tom7 that serve as the ‘trans‐binding site of the TOM complex’. These TOM elements also serve as a recruitment site for the TIM23 complex via its Tim21 subunit (Chacinska et al., 2005; Chacinska et al., 2010; Popov‐Celeketic et al., 2008; van der Laan et al., 2006). From the IMS domains of the TOM subunits, the presequence is transferred to the IMS domain of Tim50 (Geissler et al., 2002; Mokranjac et al., 2003; Schendzielorz et al., 2017; Yamamoto et al., 2002). Subsequently, the presequence is guided to the protein‐conducting half channel formed by Tim17 in a reaction that presumably involves membrane‐potential‐dependent gating of the translocase (Demishtein‐Zohary et al., 2017; Fielden et al., 2023; Sim et al., 2023). Once protruding into the matrix, the presequence is grabbed by the mitochondrial Hsp70 chaperone, which is the central element of the import motor. The binding of multiple Hsp70 chaperones drives protein translocation into the matrix in an ATP‐hydrolyzing reaction (D'Silva et al., 2004; Kang et al., 1990; Okamoto, 2002; Sato et al., 2019; Voisine et al., 1999). Finally, the presequence is recognized and processed by the MPP. All these steps have been previously described in detail in excellent reviews (Busch et al., 2023; Chacinska et al., 2009; Endo & Wiedemann, 2025; Herrmann & Bykov, 2023; Jain et al., 2025; Kizmaz et al., 2024; Ozdemir & Dennerlein, 2024).

Presequences have presumably variable efficiency in these different binding reactions, probably to fine‐tune the biogenesis of their respective precursor proteins. For example, some matrix proteins contain cysteine residues that have to be oxidized by Mia40 in the IMS. These proteins employ ‘weak’ presequences that fail to recruit the TIM23 complex to the TOM complex. They therefore use a two‐step import pathway in which they are transiently released into the IMS before finally crossing the IM (Longen et al., 2014; Peker et al., 2023). Interestingly, many mitoribosomal proteins (MRPs) follow this two‐step import mode for their translocation into the matrix. Accordingly, in poorly energized mitochondria, these MRPs are trapped in the IMS, potentially to avoid cytosolic cytotoxic problems of non‐imported precursor proteins (Flohr et al., 2025; Kang et al., 2025). This unconventional pathway—referred to as MitoTraP— might explain why many MRPs have unconventional presequences with very low prediction scores in programs such as TargetP (Bykov et al., 2022; Woellhaf et al., 2014). Once stress conditions are overcome, the IMS‐trapped proteins might either be imported into the matrix or degraded; these details of the MitoTraP pathway will still have to be uncovered.

## 5 PRESEQUENCES INFLUENCE PROTEIN (UN)FOLDING AND RECRUIT CHAPERONES

It was proposed that the presequences sterically impede the folding of precursor proteins, so that efficient folding of mitochondrial proteins is favored only after their full translocation into the matrix. According to this hypothesis, the presequence would function like a safety latch that first has to be removed by MPP cleavage before folding can commence. However, the folding of only a few precursor and mature versions of model proteins was tested experimentally, and the results of such in vitro experiments with purified proteins were heterogeneous and did not always support this idea. The presence of the presequence indeed impaired the folding of some proteins, but the folding of others was not affected (Goder et al., 1998; Murakami et al., 1990; Stoltz et al., 1995; Zhou & Weiner, 2001). Thus, the hypothesis that presequences help to maintain non‐imported precursor proteins in unfolded conformations to facilitate protein translocation is not proven experimentally yet.

In vivo, presequences might considerably influence protein (un‐)folding in the cytosol as they serve as binding sites for cytosolic chaperones. Their amphipathic structure makes them perfectly suited to be bound by Hsp70 molecules. Several cytosolic chaperone systems were found as interactors of presequences in yeast cells, such as the subunits of the nascent chain‐associated complex (NAC; Avendano‐Monsalve et al., 2022; Fünfschilling & Rospert, 1999; George et al., 1998; Ponce‐Rojas et al., 2017) and members of the Hsp70 family like Ssb1/Ssb2 (Doring et al., 2017; Dunn & Jensen, 2003). These chaperones presumably bind co‐translationally, before the general Hsp70 chaperones Ssa1/Ssa2 and their co‐chaperones Ydj1 and Xdj1 take over (Atencio & Yaffe, 1992; Becker et al., 1996; Caplan et al., 1992; Deshaies et al., 1988; Hoseini et al., 2016; Ruger‐Herreros et al., 2024).

In human cells, the Hsp70 cofactor St13 binds co‐translationally to presequences as soon as they emerge at the exit tunnel of the cytosolic ribosome (Juszkiewicz et al., 2025), facilitating the association with the Hsp70 family protein Hsc70. St13 might be identical to the presequence‐binding factor; this import‐stimulating protein was discovered more than 30 years ago, but its molecular identity was never deciphered (Murakami & Mori, 1990). The yeast homolog of St13, Sti1, was also found as an interactor of non‐imported mitochondrial proteins (Hansen et al., 2016; Hoseini et al., 2016), so the role of St13/Sti1 might be conserved. Sti1 also coordinates the interaction of Hsp70 with the Hsp90 chaperone system (Röhl et al., 2015); its specific role in the biogenesis of mitochondrial proteins, however, is still unknown.

**FIGURE 3:**
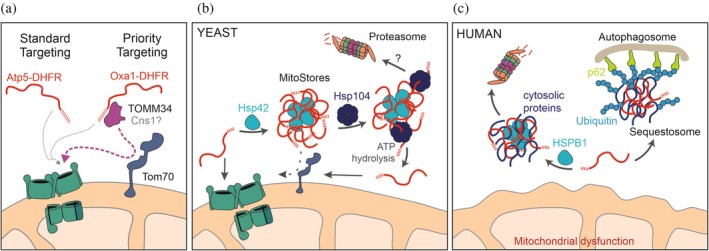
Presequences serve as binding sites for factors of the cytosolic protein quality control network. (a) Some presequences recruit TOMM34, a cochaperone of the Hsp90 system, to facilitate the interaction with Tom70. TOMM34 is present in animal cells. The yeast protein Cns1 is structurally related to TOMM34 and might play a similar role. (b) Presequences can promote the sequestration of non‐imported precursors by Hsp42 into MitoStores (Krämer et al., 2023). Hsp104 is able to remove precursor proteins from these aggregates and promote their mitochondrial import or degradation. (c) In human cells, p62 bodies might play a MitoStore‐like function in the sequestration of non‐imported precursor proteins (Amponsah et al., 2025).

The higher efficiency of longer presequences might arise from the presence of binding sites for specific components of the Hsp90 system (Hoffman et al., 2025; Rödl et al., 2025). TOMM34 (also known as Tom34) is a cytosolic co‐chaperone of Hsp90 in animal cells that supports mitochondrial protein import (Faou & Hoogenraad, 2012; Trcka et al., 2014; Trcka et al., 2020). TOMM34 is also present in reticulocyte lysate, which is used to synthesize mitochondrial precursor proteins for in vitro import assays. The high import competence of proteins synthesized in reticulocyte lysate presumably stems from the presence of factors such as TOMM34, which endow proteins with high import capacity (Figure 3a; Rödl et al., 2025). The Cns1 protein of yeast cells is structurally related to TOMM34 (Hainzl et al., 2004; Schopf et al., 2019) and might play a similar role, but its relevance for mitochondrial protein biogenesis is less clear. Hsp90 (with the assistance of TOMM34/Cns1) presumably directs precursors to Tom70 on the mitochondrial surface to promote their unfolding and efficient translocation into mitochondria (Backes et al., 2018; Backes et al., 2021; Koch et al., 2024; Komiya et al., 1997; Rödl et al., 2025; Yamamoto et al., 2009; Young et al., 2003).

## 6 PRESEQUENCES CAN INDUCE THE INCORPORATION INTO MitoStores

Non‐imported mitochondrial precursor proteins can be sequestered into cytosolic aggregates called MitoStores (Bertgen et al., 2024; Kaushik et al., 2025; Krämer et al., 2023; Nowicka et al., 2021; Schlagowski et al., 2021). They were called MitoStores because—upon inhibition of the mitochondrial import machinery—these structures are largely composed of mitochondrial precursor proteins. However, comparable foci were reported under different stress conditions and called Q bodies or cytoQs. From a biochemical perspective, all these aggregates might be identical and just differ in the composition of their client proteins.

In yeast cells, small heat shock proteins, in particular Hsp42, serve as nucleation factors that are necessary for MitoStore formation. The involvement of small heat shock proteins in the formation of different types of aggregates has been well established (Grousl et al., 2018; Haslbeck et al., 2019; Haslbeck et al., 2020; Miller et al., 2015; Mogk & Bukau, 2017). The disaggregase Hsp104 can release precursor proteins from MitoStores to promote their import. Only a sub‐group of newly synthesized mitochondrial proteins is included in MitoStores, and the presence of presequences is critical for their association (Figure 3b; Krämer et al., 2023). Thus, presequences serve as an entry ticket for MitoStores, but the underlying structural features that promote integration into MitoStores are not yet known. Although precursor sequestration into MitoStores is protective, such cytosolic aggregates can become toxic upon prolonged stress conditions (Nowicka et al., 2021; Sontag et al., 2017; Wrobel et al., 2015).

The formation of MitoStore‐like cytosolic aggregates that contain mitochondrial precursor proteins was also observed in human cells (Kravic et al., 2018). For example, the unbalanced gene expression in aneuploid cells induces cytosolic aggregates that contain many mitochondrial proteins (Amponsah et al., 2025; Amponsah & Storchova, 2025). These structures are bound by p62 (also called SQSTM1 or sequestosome 1) and are referred to as p62 bodies. Like MitoStores, these p62 bodies contain small heat shock proteins (HSPB1) as seeds, are of transient nature, and can be resolved over time, either by the release of individual proteins or by autophagy (Figure 3c).

## 7 PRESEQUENCES CAN SERVE AS DEGRONS TO CONTROL PROTEIN STABILITY

If their import fails, most non‐imported mitochondrial precursor proteins are rapidly degraded by the proteasome. Only a few of such precursor proteins, like that of Ilv2, escape proteasomal degradation, presumably to carry out some function in the cytosol or nucleus (Pines et al., 2024; Shakya et al., 2021).

**FIGURE 4:**
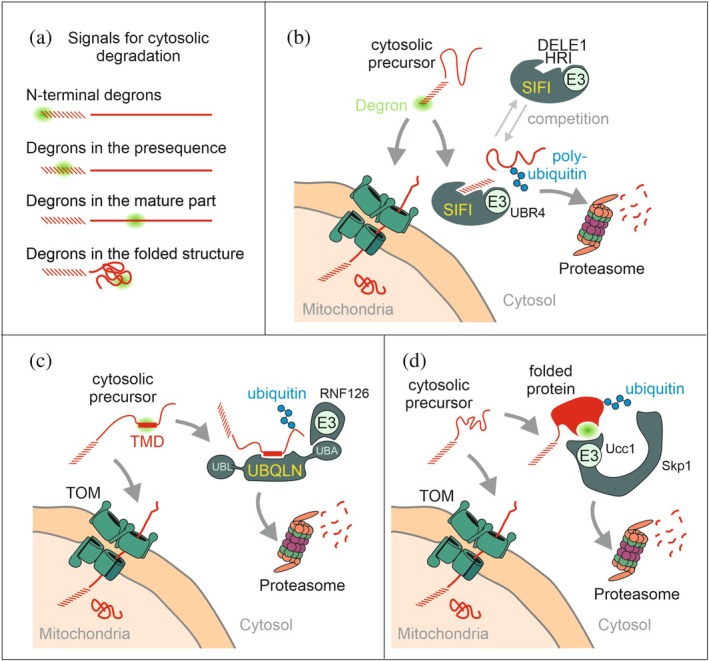
Segments in the presequence and mature part of precursors can promote degradation. (a) Schematic representations of the positions of potential degrons in different regions of mitochondrial precursor proteins. (b) The human ubiquitin ligase complex SIFI binds presequences of mitochondrial precursors and facilitates ubiquitination of the related proteins (Haakonsen et al., 2024; Yang et al., 2025). SIFI also promotes the degradation of DELE1 and HRI, which serve as components of the integrated stress response. The accumulation of precursors in the cytosol prevents the degradation of DELE1 and HRI so that the integrated stress response can commence. (c) Ubiquilin (UBQLN) binds to the transmembrane domains (TMDs) of mitochondrial inner membrane proteins in the cytosol and promotes their ubiquitination by the ubiquitin ligase RNF126. Thus, ubiquilin serves as a triage factor that promotes either import or, if this fails, the degradation of precursor proteins (Itakura et al., 2016). (d) Ubiquitin ligases such as the F‐box protein Ucc1 can also specifically recognize the folded states of (potentially toxic) mitochondrial precursor proteins to purge their activity from the cytosol (Hayashi et al., 2024). This process prevents the accumulation of the citrate synthase Cit1 in the cytosol of yeast cells.

In yeast, several E3 ubiquitin ligases, including San1, Ubr1, Doa10, and Rsp5, cooperate in mediating the degradation of non‐imported mitochondrial precursor proteins (Kowalski et al., 2018; Metzger et al., 2020; Schulte et al., 2023; Shakya et al., 2021). Segments in the presequence or in the mature part of the precursors might serve as degrons and promote precursor ubiquitination by these E3 proteins (Figure 4a). However, detailed studies are still missing.

In humans, presequences of non‐imported precursor proteins are recognized by a large complex called SIFI (for silencing factor of the integrated stress response). This complex contains the ubiquitin ligase subunit UBR4, which ubiquitinates its substrates and promotes their subsequent proteolytic degradation (Figure 4b; Grabarczyk et al., 2025; Yang et al., 2025). The SIFI complex plays a central role in the response of cells to stress arising from mitochondrial import defects: upon import problems, DELE1, a protein that is normally fully translocated into mitochondria, becomes cleaved by the mitochondrial protease Oma1 and released into the cytosol. Cytosolic DELE1 stabilizes the kinase HRI (heam‐regulated inhibitor kinase), which then phosphorylates the initiation factor eIF2α and thereby curtails protein synthesis (Ahola et al., 2022; Fessler et al., 2020; Fessler et al., 2022; Guo et al., 2020; Haakonsen et al., 2024; Rivera‐Mejias et al., 2023). The reduced translation caused by mitochondrial dysfunction is one facet of the integrated stress response and allows cells to overcome problems related to mitochondrial dysfunction. Interestingly, DELE1 and HRI are also substrates of SIFI but remain stable upon import failure as the non‐imported precursor proteins competitively inhibit their degradation (Haakonsen et al., 2024; Yang et al., 2025). However, once mitochondria recover and start to import precursor proteins again, SIFI becomes free to mediate the degradation of the cytosolic pools of DELE1 and HRI, thereby deactivating the integrated stress response.

Not only the presequence but also segments in the mature region of precursor proteins determine their proteolytic stability. For example, hydrophobic transmembrane domains in non‐imported precursor proteins are bound by ubiquilin, which recruits the ubiquitin ligase RNF126 to facilitate the ubiquitination of ubiquilin‐bound precursors (Figure 4c; Itakura et al., 2016; Liu et al., 2025). Ubiquilin thereby serves as a triage factor to reduce the risk of toxic effects arising from non‐imported mitochondrial membrane proteins. The yeast homolog of ubiquilin Dsk2 might play a similar role, even though its involvement is less clear (Schulte et al., 2023). Dsk2 cooperates with the peptidyl‐tRNA hydrolase Pth2 in the degradation of non‐imported proteins (Bertram et al., 2025; Ishii et al., 2006).

The cytosolic species of some proteins that are dually localized to mitochondria and cytosol lack their presequence. The mature cytosolic forms of these proteins are generated after import of the N‐terminal region of the molecule into mitochondria; upon MPP cleavage, these translocation intermediates move back into the cytosol by reverse translocation (Stein et al., 1994). In the case of the citrate synthase Cit1, failure of its import into mitochondria gives rise to folded, functionally active citrate synthase in the cytosol (Nakatsukasa et al., 2015; Nishio et al., 2023). Such cytosolic citrate synthase is detrimental due to metabolic poisoning. To avoid this, the ubiquitin ligase complex Skp1‐Cdc53‐Ucc1 recognizes the folded Cit1 in the cytosol and promotes its ubiquitination and degradation even without contribution of a presequence (Figure 4d). The folding of Cit1, which is induced by a Hsp70 (Ssa1) and its J‐protein (Ydj1), is a prerequisite for the recognition by the ubiquitin proteasome system (Hayashi et al., 2024; Nishio et al., 2023).

## 8 FINAL REMARKS

Recent studies demonstrate that mitochondrial presequences are more than entry tickets that direct proteins into mitochondria. They interact with multiple factors that promote the targeting, (un)folding, and proteolytic stability of the newly synthesized proteins. In addition, presequences might also determine whether proteins are imported co‐ or post‐translationally; but details still wait to be deciphered (Luo et al., 2025; Zhu et al., 2025).

Their very transient presence—most presequences are removed by MPP very quickly after their initial synthesis due to a fast mitochondrial import—has so far inhibited the identification of binding partners and special motifs. Some recent developments of novel methods, such as proximity labeling, site‐specific crosslinking, usage of cell free translation systems, and in vivo import assays might overcome these problems. The employment of these novel approaches, and others to be discovered, will shed new light on the complex physiological functions of presequences.

## AUTHOR CONTRIBUTIONS

**Erik Marcel Heller:** Conceptualization; writing – review and editing; visualization. **Svenja Lenhard:** Conceptualization; writing – review and editing; visualization. **Doron Rapaport:** Conceptualization; writing – review and editing; funding acquisition. **Johannes Herrmann:** Conceptualization; writing – original draft; visualization; funding acquisition.

## Data Availability

Data sharing not applicable to this article as no datasets were generated or analyzed during the current study.
